# Challenges in the working relationship between professional nurses and clinical associates in selected district hospitals in South Africa

**DOI:** 10.4102/hsag.v28i0.1927

**Published:** 2023-04-28

**Authors:** Emmah M. Mokoena, Tinda Rabie, Antoinette du Preez

**Affiliations:** 1 NuMIQ Focus Area, School of Nursing Science, Faculty of Health Sciences, North-West University, Potchefstroom, South Africa

**Keywords:** professional nurses, clinical associates, professionalism, professional relationships, service delivery

## Abstract

**Background:**

Clinical associates were introduced in South Africa to address physician shortages in healthcare. Professional relationships between physicians and professional nurses (PNs) have been widely researched, but none specifically between the new cadre of clinical associates and PNs.

**Aim:**

This study aimed to understand the professional working relationship between PNs and clinical associates.

**Setting:**

Selected district hospitals within Mpumalanga Province, South Africa.

**Method:**

A qualitative descriptive design was used. Professional nurses were purposely sampled, and an all-inclusive sampling method was used for clinical associates in selected district hospitals within Mpumalanga Province, South Africa. Twelve (*N* = 12) semi-structured, individual interviews (PNs *n* = 6; clinical associates *n* = 6) guided by an interview guide were conducted in English. The interviews were audio recorded and transcribed verbatim by an independent transcriptionist. Tesch’s eight steps of data analysis were employed to analyse the data. An independent co-coder assisted with data analysis.

**Results:**

This study yielded four themes: (1) professional relationship defined, (2) professional relationship characteristics, (3) professional challenges applicable to both PNs and clinical associates and (4) personal professional challenges applicable to clinical associates only.

**Conclusion:**

This study demonstrated that the professional relationships between PNs and clinical associates are affected by various challenges, which could be resolved within the department through in-service training and good communication.

**Contribution:**

This is one of the first studies that highlight the professional relationship challenges between PNs and clinical associates.

## Introduction

Globally, healthcare professionals (HCPs) are considered to be the backbone of the healthcare system (Rabie, Klopper & Coetzee 2017). Professional relationships between them can be viewed as a cornerstone for effective patient care and service delivery. Professional relationships in healthcare are often associated with patient–practitioner relationships; however, collegial professional relationships between HCPs are often overlooked (Nakhaee & Nasiri 2017). These relationships with colleagues have their unique demands and concerns and may affect the care of patients. Literature has shown that professional collegial relationships between the HCPs are very important in maintaining optimal health service delivery. Therefore, understanding of training, scope of practice and expertise of other HCPs in the multidisciplinary team is of utmost importance and could be achieved through successful interprofessional education and interprofessional collaboration (Bollen et al. 2019; Hepp et al. 2014; Karam et al. 2018; Reeves et al. 2014). However, according to the authors’ knowledge, currently, no literature is available on the professional working relationship between professional nurses (PNs) and clinical associates (CAs).

South Africa forms a part of low-income and middle-income countries. In comparison to high-income international countries, shortage of physicians is a problem and is on a larger scale when calculating the ratios of physicians to patients. South Africa averages 60 physicians to 100 000 patients, compared to world average of 152 physicians to 100 000 patients (Rabie, Klopper & Watson 2016). To address this problem, the South African government proposed the establishment of more medical schools to educate and train additional physicians (Bateman 2013). However, the establishment of more medical schools has not yielded the expected outcome as the number of physicians trained yearly is still insufficient. South Africa’s medical schools graduate about 1800 students a year, and comparing this to the 1970s where about 1200 students graduated, it shows that the shortage has gotten worse over the years. This is because the population has more than doubled from 24.3 million in the mid-1970s to about 56.5 million (Veller 2018). This will add to the ageing population, which will require more treatments for chronic ailments in future. And the one solution to counteract the shortage of physicians in South Africa was to introduce CAs into the healthcare system as their training was less costly and time-intensive than that of physicians (Cawley & Hooker 2018). The South African government followed international trend by introducing a new cadre of HCPs forming part of the clinical team working under the supervision of a physician (Moodley et al. 2014). Thus in 2004, the National Task Team was commissioned to develop the Bachelor of Clinical Medical Practice (BCMP) as an education and training programme for CAs. The education and training of these CAs were based on generalist rather than specialist skills (Doherty et al. 2013). In 2017, as many as 920 graduated CAs were stationed in hospitals throughout South Africa (Bert 2013).

Although PNs, also known as registered nurses, can also be identified as generalists, they enjoy a more independent scope of practice compared to CAs. The Nursing Act defines a registered nurse’s scope of practice as follows: PNs are responsible for ‘diagnosing a health need and prescribing, providing and executing a nursing regimen to meet the needs of patients’ (Esterhuizen 2016:13). Clinical associates, on the other hand, adhere to a limited scope of practice and must function under the direct supervision of a physician. The CAs scope of practice is tailored to the specific context and unique needs of district hospitals (Doherty et al. 2012). Furthermore, the CAs are expected to apply their skills in consultation, physical examination and counselling (Doherty et al. 2012). The CAs were not introduced to substitute PNs or physicians; although the scope of practice is clearly specified, it seems that confusion surrounding the CAs scope of practice emerged. Owing to the introduction of a new occupation in multidisciplinary healthcare team, both CAs and PNs were not very clear on each other’s roles in the practice environment (Cawley & Hooker 2018).

The CAs are a new mid-level category of HCPs with the skills and knowledge to function effectively, mainly in district hospitals. They undergo 3 years of full-time training at university level to obtain a degree in BCMP qualification. Regulations regarding their scope of practice were gazetted and signed by the Minister of Health in 2016, stipulating that a clinical associate may perform allocated medical acts under the supervision of a physician in accordance with their training. Their scope of practice entails assessing patients, prescribing treatment and performing minor surgeries under the supervision of a physician.

Professional nurses primarily train for a 4-year diploma at nursing colleges or a 4-year degree at universities, with an opportunity to specialise in a variety of specialities in nursing. Professional nurses are guided by their scope of practice based on their field of specialisation. Professional nurses are expected to comply with policies and legislation governing their profession so that they are able to identify complications and intervene accordingly in order to deliver professional care (Maputle & Hiss 2010:5).

The World Health Organization (2017) advocates that those members of the multidisciplinary healthcare team should be guided by a code of ethics, which includes a commitment to professionalism. In this regard, professionalism is characterised by autonomous, evidence-based decision-making by members of an occupation who share similar values and education (Nursing and Midwifery Council 2015; World Health Organisation 2017). As aforementioned, it is clearly important that professional working relationships between PNs and CAs should be understood to ensure professionalism in the practice environment.

### Aim

This study aimed to gain an understanding of the professional working relationship between PNs and CAs.

### Objective

To describe the challenges of the PNs and CAs regarding their working relationship in the selected district hospitals.

## Research methods and design

### Study design

A descriptive qualitative design was used to gain a better understanding of the challenges in the working relationship between the PNs and CAs. The design gives a description of how PNs experienced the professional relationship with the CAs, followed by a similar description of the CAs experiences. It provided a straightforward description of experiences and perceptions in an area where little is known about the topic under investigation (Sandelowski 2010). This design was therefore deemed most appropriate as it recognises the subjective nature of the challenge and the different experiences of the participants (Bradshaw et al. 2017).

### Setting

The study setting was initially supposed to take place in seven selected district hospitals in a district, Mpumalanga Province, but only PNs and CAs from four district hospitals agreed to participate in the study. The departments of interest were the outpatient, casualty and theatre departments. These departments generally have the highest patient load within the district hospitals, and it is where CAs mainly work. Other departments in general have no CAs working there. District hospitals provide 80% of surgical care and basic life-saving procedures (Bentounsi et al. 2021).

### Study population and sampling strategy

The study population consisted of (*N* = 16, *n* = 6) PNs and (*N* = 12, *n* = 6) CAs. Purposive sampling was used because it assisted the authors to solely select the participants who can address the aim of the study. Both populations had to meet the following inclusion criteria: both PNs and CAs had to practice in the identified district hospitals, work in the outpatient, casualty and theatre departments, English literate and be willing to participate in the study. Exclusion criteria for the PNs included any nurse who is not a PN, unit managers working at outpatient, casualty or theatre department, and PNs who has never worked with CAs. Exclusion criteria for CAs included student CAs and CAs working in primary healthcare setting.

### Data collection

Data were collected between September 2017 and May 2018. After obtaining ethical approval from the North-West University, a letter seeking permission to carry out the study was submitted to the Head of Research and Ethics Committee, provincial Department of Health in Mpumalanga Province. After obtaining approval from the Head of Research and Ethics Committee, a letter was sent to the chief executive officers of each hospital asking permission to conduct the study. The first author arranged a one-on-one meeting with the gatekeepers; matrons for the PNs and clinical managers for the CAs. The authors were not staff members at any of the selected hospitals.

#### Pilot study

A pilot study was done which included one PN and one CA to ensure the interview questions were clear and that the aim of the study could be reached (Kallio et al. 2016). The results of the pilot study indicated that no adaptions were needed as all the participants understood the questions and data revealed the questions answered the aim of the study. The pilot study participants were excluded from the main study. The questions of the interview guide were developed by the authors and included questions related to the aim and objectives of the study (see Table 1). The interview guide was validated by the pilot study; the results of the pilot study revealed rich, descriptive account of the study topic as well as the participants’ willingness to elaborate on the topic. The interviews lasted approximately 45–60 min and were audio recorded.

**TABLE 1:** Interview guide.

Interview guide
What is your view on the concept ‘professional relationships’? What is your view on the professional relationships between professional nurses and clinical associates? What in your view is the role of clinical associates in the practice environment? What in your view is the role of professional nurses in the practice environment? In your opinion, what do you think can professional nurses do to improve the professional relationships with clinical associates? In your opinion, what do you think can clinical associates do to improve the professional relationships with professional nurses?

### Data analysis

The demographic data were entered onto an Excel spreadsheet and the audio recordings were transcribed verbatim immediately after the interviews by an independent transcriptionist. The data were analysed using Tesch’s steps of content analysis with the assistance of an independent co-coder (Creswell 2013). The first author and co-coder coded the data independently. No software was used during data analysis. All data collected through interviews, voice recorder and observations were transformed into written text with the date, time and the place where the interview took place. Tesch’s eight steps of data analysis as outlined in Botma et al. (2010:223) are as follows:

- Step 1: All data collected through interviews, digital recorder and observations were transcribed into written text with the date, time and place where the interview took place.
- Step 2: The data were read through several times to obtain a general sense of the information and to reflect on its overall meaning.
- Step 3: Several transcripts were read through and highlighted or had segments (phrases) in them.
- Step 4: A list was compiled of all the topics that came to mind.
- Step 5: Compiled list was checked to analyse the transcripts by looking for segments (phrases) from the transcripts that fit the topics.
- Step 6: All the segments that fitted a particular topic were put together and given descriptive names as subthemes.
- Step 7: Subthemes were sorted and grouped together, then given descriptive names as themes.
- Step 8: Recordings were done.

### Ethical considerations

Ethical approval was granted by the Health Research Ethics Committee at the North-West University (reference number NWU-00307-20-A1; 11 Sept. 2017) in South Africa. Ethics approval was also obtained from the Head of Research and Ethics Committee, Provisional Department of Health in Mpumalanga Province, and the Department of Health, Head of Research and Ethics Committee. A letter was sent to each of the Chief Executive Officers of the hospitals to conduct this study; all seven hospitals gave permission.

No harm was imposed to the participants during the study. Participation was voluntary. An information letter containing the objectives, purpose and benefits of the study was given to the participants prior to their signing of the informed consent. Permission to audio record the interviews was sought and granted by the participants. The interviews took place in private rooms where the participants worked. Privacy and confidentiality were ensured by making sure that the participants’ names were not mentioned in any of the data collected and nowhere in the description of the findings. The transcripts and audio recordings were locked away. Subsequently, the participants were assured that they may withdraw from the interview at any stage, without repercussions.

## Measures of trustworthiness

Trustworthiness was ensured using Lincoln and Guba criteria of credibility, transferability, dependability and conformability. A qualitative study is deemed credible when it provides an accurate interpretation and description of human experience. *Credibility* was enhanced by using verbatim transcription, prolonged engagement with the participants and field notes which added nonverbal cues such as body language. *Transferability* was achieved by focusing on a representative population and by providing description of the research method and study context in which the study took place. By using an interview guide, the same questions could be directed to all the participants. *Dependability* was maintained by appointing a qualitative independent co-coder and engaging the supervisors to ensure the results are a true reflection of the interviews. *Conformability* was performed by reaching the consensus on the findings of study with the independent co-coder.

## Results

### The participants

The participants consisted of four females and eight males, between the ages of 20 and 40 years. There were six PNs and six CAs.

### Themes and subthemes identified

Three themes and six subthemes were identified from the data of the PNs. The themes were professional relationship defined, professional relationship characteristics and professional relationship challenges (see Table 2). Four themes and 10 subthemes were identified from the data of the CAs. These themes comprised professional relationship defined, professional relationship characteristics, professional relationship challenges and personal professional challenges (see Table 3).

**TABLE 2:** Themes and subthemes arising from the professional nurses’ data.

Themes	Subthemes
1. Professional relationship defined	1.1 Relationship among colleagues
2. Professional relationship characteristics	2.1 Positive characteristics 2.2 Negative characteristics
3. Professional relationshipchallenges	3.1 Attitude 3.2 Functional challenges related to clinical associates 3.3 Ministerial, interdepartmental and interprofessional collaboration

**TABLE 3:** Themes and subthemes arising from the clinical associates’ data.

Themes	Subthemes
1. Professional relationship defined	1.1 Collegial relationships
2. Professional relationship characteristics	2.1 Positive characteristics
3. Professional relationship challenges	3.1 Attitude 3.2 Functional challenges related to clinical associates 3.3 Functional challenges related to professional nurses 3.4 Ministerial collaboration
4. Personal professional challenges	4.1 Lack of independence 4.2 Poor remuneration 4.3 Lack of career advancement 4.4 Supporting profession to physician shortages

The data of the PNs will firstly be presented and thereafter those relevant to the CAs.

### Themes and subthemes identified from the professional nurses’ data

Themes and subthemes derived from the clinical PNs’ data are discussed in Table 2.

#### Theme 1: Professional relationship defined

The results highlighted that there was no single or a specific way of defining professional relationship. Rather it is a concept that can be defined in different ways and mean different things to different people in different contexts.

**Subtheme 1.1: Relationship among colleagues.** Data highlighted that there was no single definition of professional relationship; rather it is a concept that can be defined in different ways by different people in different contexts. Some of the participants defined a professional relationship as a relationship between PNs while others included other HCPs in the description. The PNs said:

‘A professional relationship is what us as colleagues or people who are working together.’ (PNP1, male, 40 years)

‘Relationship between the other healthcare professionals.’ (PNP2, male, 33 years)

Professional relationship is important in the practice environment as it enhances relations between different professions for the betterment of the service delivery.

#### Theme 2: Professional relationship characteristics

Professional relationship characteristics are fundamental in the practice environment.

**Subtheme 2.1: Positive characteristics.** Some of the PNs portrayed positive characteristics such as giving support to each other, open communication regarding their uncertainties and teamwork in terms of working together to achieve optimal health service delivery as essential elements of a professional relationship between the two groups. They said:

‘Support each other in everything that we do. I believe that once we have a strong support system as a team, that we … there’s nothing that we cannot conquer.’ (PNP4, female, 38 years)

‘So, we need to sit down and talk and leave our pride aside.’ (PNP6, male, 33 years)

‘We should be able to work together as a team … be able to build on each other.’ (PNP4, female, 38 years)

**Subtheme 2.2: Negative characteristics.** Some of the participants highlighted the negative characteristics such as lack of respect by the CAs and not adhering to the timings of the practice environment. They believed that they cannot trust the CAs, that they were not respected and that the CAs did not behave in a professional manner:

‘They [*clinical associates*] come, they go out and do everything that they’re doing outside there that we don’t know and that is a problem, we cannot trust them.’ (PNP1, male, 40 years)

‘It clearly shows that there’s no respect because my profession is not respected here.’ (PNP1, male, 40 years)

‘I [*professional nurse*] can’t be professional because it’s weighing down on me. So, I feel that they [*clinical associates*] should decide where they stand … and then be professional about the work.’ (PNP4, female, 38 years)

#### Theme 3: Professional relationship challenges

Three subthemes that PNs identified to define professional relationship challenges were attitude, functional challenges related to CAs, and ministerial, interdepartmental and interprofessional collaboration.

**Subtheme 3.1: Attitude.** Some of the PNs believed the CAs displayed a poor attitude such as the CAs looking down on the PNs. Attitude, whether good or poor (bad), is very important as it is beneficial to the practice environment as well as individual employees. Good attitude improves efficiency of communications, better teamwork and collaboration.

This was described as:

‘Yes, there is an attitude … bad attitude from them, towards nurses.’ (PNP2, male, 33 years)

‘I [*professional nurses*] feel it’s either they [*clinical associates*] need to change their attitude to become more positive.’ (PNP4, female, 38 years)

**Subtheme 3.2: Functional challenges regarding the clinical associates.** The PNs described various functional challenges in terms of their professional relationships with the CAs. This included a lack of designation, seniority, uncertainties about the scope of practice, lack of independence and role clarification in the practice environment. A lack of designation was described as:

‘The doctors themselves, they don’t understand ’cause they ended up themselves calling them [*clinical associates*], “Doctor so and so come and assist me here.” They don’t have a real designation.’ (PNP5, female, 35 years)

‘Even other patients when they leave the hospital, will say “I was seen by the doctor” but while they were seen by the clinical associate. It means they don’t introduce themselves as clinical associates.’ (PNP2, male, 33 years)

Relationship problems in terms of seniority, lack of independence and role clarification and scope of practice were described as:

‘Yes, there is a problem when it comes to seniority … they are like seniors to me whereas they cannot even do anything on their own and that is the problem that we are facing as professional nurses.’ (PNP1, male, 40 years)

‘What is their [*clinical associates*’] scope of practise? I have never seen it … when you ask them … one of them … some will say it’s been under review, but no one has ever physically seen the scope of practise in the file.’ (PNP4, female, 38 years)

‘A medical doctor must sign, it cannot go alone, that prescription, without being countersigned by a real medical doctor.’ (PNP5, female, 35 years)

**Subtheme 3.3: Ministerial, interdepartmental and interprofessional collaboration.** Most PNs pointed out that the Minister of Health and departments in the district hospitals should provide more information about the introduction of CAs. Such clarification could have been provided through the media, workshops, roadshows and in-service training.

‘Minister must start to use things like media, workshops and roadshows, and there must be a task team that is appointed to go around South Africa, informing people about clinical associates.’ (PNP1, male, 40 years)

‘The department … if they can provide … if they may provide full information about their scope of practice, of clinical associates to be visible, transparent, known by everyone at the facility, and then when they come, the department needs to do in-service training to the nurses, informing them about the clinical associates.’ (PNP2, male, 33 years)

### Themes and subthemes arising from the clinical associate’s data

Themes and subthemes derived from the CAs data are discussed in Table 3.

#### Theme 1: Professional relationship defined

Professional relationship cannot be defined without mentioning collegial relationships which are among the most prevalent types of interpersonal relationships. Collegial relationships are important in producing positive outcomes in the practice environment.

**Subtheme 1.1: Collegial relationship.** The CAs defined this relationship in various terms and did not indicate who colleagues were. However, they were also of the opinion that this role should be beneficial to the patients. They said:

‘A professional relationship is an ongoing interaction between two people … We must work together professionally to help, to win the goal, which is the patient.’ (CAP2, female, 24 years)

‘Professionalism, for example colleague to colleague, they can have a good relationship, doctor-to-patient – they might have a professional relationship.’ (CAP3, male, 29 years)

#### Theme 2: Professional relationship characteristics

Positive professional relationship characteristics that derived from the data are discussed.

**Subtheme 2.1: Positive characteristics.** Only positive professional relationship characteristics were identified by the CAs, namely mutual respect and support:

‘Respect those people that you work with, they [*professional nurses*] will respect you back.’ (CAP5, male, 26 years)

‘They [*professional nurses*] are supportive if you’ve got a query then you go and ask them and then they’ll assist you.’ (CAP6, male, 25 years)

Mutual respect and support promote effective teamwork. An increase in respect helps to improve communication between the HCPs.

#### Theme 3: Professional relationship challenges

Professional relationship challenges can affect the morale and have negative impact on the health service delivery. Attitudes formed by misunderstandings have been argued to affect interpersonal relationships in healthcare systems.

**Subtheme 3.1: Attitude.** Some of the CAs felt that it is difficult to work with the PNs because of their attitude. Their experience of this continuing poor attitude resulted in one wanted to retaliate.

‘But instead, they’re [*professional nurses*] giving us attitude, they’re difficult to work with … they undermine us, they say we’re acting as doctors, the reason being we are the ones who work closely with the doctors whereas them they work behind the scenes.’ (CAP2, female, 24 years)

‘It’s about our job but if you’re [*professional nurses*] continuing to give us such a bad attitude, I think I’ll have to retaliate also.’ (CAP3, male, 29 years)

**Subtheme 3.2: Functional challenges related to clinical associates.** The CAs identified various functional challenges related to their roles. These challenges were lack of designation, seniority and role clarification in the practice environment and uncertainty about the scope of practice. The participants described the role confusion as follows:

‘Patients tend to become confused; when they see us, they’ll be like “doctors,” so we [*clinical associates*] don’t know how to correct them. So, we need that profession to be also put into the picture.’ (CAP2, female, 24 years)

‘Yes, we do have hope if only our [*clinical associates*’] roles can be clarified.’ (CAP2, female, 24 years)

Some of the CAs were of the option that their qualifications were superior to that of a PN and one said:

‘What I can say … [*I have a more*] advanced qualification than the nurses, so … yes, our relationship is not yet balanced.’ (CAP3, male, 29 years)

Some were not clear of their scope of practice while others were not sure who should inform the nurses about their scope:

‘Another problem we [*clinical associates*] don’t have a scope.’ (CAP3, male, 29 years)

‘So, who’s responsible is it for letting nurses know what my scope of practise is, is it me or the head of the department or the clinical manager.’ (CAP5, male, 26 years)

**Subtheme 3.3: Functional challenges related to professional nurses.** The CAs identified various functional challenges relating to the PNs. This included sharing of experience, giving advice and guidance, seniority and conflict in the practice environment. They said:

‘Nurses should make sure that they share their experiences with us.’ (CAP2, female, 24 years)

‘There is no good relationship because they [*professional nurses*] think they’re better qualified than these ones [*clinical associates*] so that’s why there’s always a clash there at workplace.’ (CAP3, male, 29 years)

**Subtheme 3.4: Ministerial collaboration.** Some of the CAs felt that not enough has been done, especially by the Minister of Health, to introduce them to the public healthcare system.

‘The Minister didn’t introduce this course very well.’ (CAP6, male, 25 years)

‘So if our minister, our current minister can introduce us properly to the system and register us as … like nurses who have been long there we’ll get along together and the working environment will be of the right state.’ (CAP2, female, 24 years)

#### Theme 4: Personal professional challenges

The conditions of practice from the regulations defining the scope of practice of CAs state that a clinical associate may not conduct an independent practice and must work under the supervision of a physician identified by the service in which the clinical associate is working.

**Subtheme 4.1: Lack of independence.** The participants highlighted their lack of independence and that they must practice under the direct supervision of a medical practitioner was seen as a hindrance as they believed they could practice independently.

‘They wrote all the procedures but they wrote “under supervision,” … even for oxygen they write at the top it’s under supervision. There are certain skills I believe we [*clinical associates*] should do without any supervision.’ (CAP6, male, 25 years)

‘I can prescribe but it needs to be counter-signed.’ (CAP5, male, 26 years)

‘Cause they [*physicians*] always countersign, the senior physician countersign.’ (CAP1, female, 29 years)

**Subtheme 4.2: Poor remuneration.** The CAs believed that they were poorly remunerated. Some believed their dependent role influenced their remuneration. They felt that their salaries did not reflect the kind of work they were doing daily. They said:

‘I don’t know how to explain it, but we’re really underpaid.’ (CAP6, male, 25 years)

‘We didn’t get gradings or whatever they call it, we just stuck … even the salary, cause a clinical associate who started working six or five years ago, still get the same salary I get so.’ (CAP1, female, 29 years)

**Subtheme 4.3: Lack of career advancement.** Lack of career advancement was also evident as many CAs felt that they are stagnant because of career advancement as there are no promotion opportunities and no plans in place for them regarding their career path.

‘Once you are a clinical associate, like I’ve been qualified since 2012, nothing different has changed. Nothing different has changed. Still, it’s the same way. We don’t have senior clinical associates, chief clinical associates, things like that.’ (CAP4, male, 32 years)

‘There is no improvement, career-wise.’ (CAP3, male, 29 years)

**Subtheme 4.4: Supporting profession to physician shortages.** Some of the CAs believed their occupation is not valued as it was merely created to fill the gap related to the shortage of medical practitioners. They said:

‘It’s to fill the gap, the void created by the shortage of doctors.’ (CAP3, male, 29 years)

‘So, there is a huge gap for doctors. So … as this course was introduced, it was to just patch on the gap.’ (CAP6, male, 25 years)

## Discussion

The study provided evidence that both populations, the PNs and CAs, understood what a professional working relationship entails. However, irrespective of this knowledge, the relationships between the two groups were riddled with challenges. The CAs functional challenges included lack of designation, seniority, role clarification, lack of independence and knowledge about the scope of practice of the CAs and physician shortages identified by both the PNs and the CAs.

Clinical associates form part of the collaborative clinical team (Doherty et al. 2013) and assist physicians by relieving their workload, thus allowing them to focus on more complex cases. As a result, patients can be treated sooner. However, a specific challenge highlighted by both the PNs and CAs was that CAs are unsure about their scope of practice. These findings are concerning because CAs scope of practice includes certain surgeries, developing treatment plans, ordering and/or performing diagnostic and therapeutic procedures and prescribing medications (South Africa 1974). As PNs and CAs work together, it is important that nurses must be clear about their colleagues’ scope of practice to ensure role clarification in the practice environment.

Functional challenges related to PNs included that the CAs felt that PNs should share their professional expertise, give advice and provide guidance. Information and knowledge are valuable resources and keys to success – also in the healthcare system (Khanum et al. 2016). Furthermore, both populations identified a seniority complex as a challenge, mostly because of hierarchical attitudes in the practice environment, potentially leading to counterproductive actions. A further challenge was identified, namely that conflict in the practice environment hinders service delivery and impedes professional relationships. One of the reasons pointed out for conflict in this environment is the difficulty to incorporate CAs within the South African Health System (Mgobhozi 2019). This issue was not foreseen when the curriculum was developed for this cadre.

The difficulty in incorporating the CAs within the South African Health System causes negative professional characteristics in the relationships such as distrust, disrespect and unprofessionalism. Disrespectful behaviour hampers communication and collaboration, causing low staff morale and an unhealthy and hostile practice environment (Grissinger 2017), which may lead to distrust and disrespect. Disrespect can be countered by forming a committee of HCPs in respective district hospitals, consisting of members of the multidisciplinary team (Grissinger 2017), even though it cannot be said to be a certainty that it will yield positive outcomes. In the authors’ opinion, such a committee could draw up a code of professionalism, establish a standard and assertive communication process, train staff and implement programmes such as conflict management, team building projects and workshops on professionalism.

However, some PNs and CAs also elicited positive relationship characteristics such as mutual respect, support, open communication and teamwork, which were opposite to the negative professional characteristics. According to Dinndorf-Hogenson (2015), these positive relationship characteristics are vital because mutual respect goes hand in hand with effective communication to enhance teamwork. Communication and teamwork can also be improved when HCPs show mutual acceptance and do not attempt to dominate each other within the practice environment (Okuyama, Wagner & Bijnen 2014).

Furthermore, collaboration was found to be important when introducing CAs into the healthcare system. Such collaboration could improve professional networking and education as well as enhance public health programmes (Couper 2014). Participants also pointed out that National Department of Health collaboration is necessary yet not continually present. This finding is in line with other authors, namely that the backing of the Ministry of Health cannot always be considered a supportive factor (Couper & Hugo 2014). Collaboration with the Ministry of Health would provide crucial support by introducing HCPs and the community to the role of CAs. Such community awareness could be created through available channels, for example, the media, workshops and roadshows.

Interdepartmentally, hospital management can initiate relevant in-service training, and collaboration could be improved through communication using meetings and disclosing all HCPs’ scope of practice, especially those of CAs. Interprofessional collaboration is when multiple healthcare workers from different professional backgrounds work together with patients, families and communities to deliver the highest quality of care when collaborators combine their skills and talents to deliver quality patient care by engaging and sharing perspectives (Janssen et al. 2017).

Interprofessional collaboration through in-service training can focus on defining professional relationships, how to enhance the positive aspects thereof, how to rectify negative characteristics of a professional relationships and how to deal with the challenges.

In terms of their scope of practice, CAs are allowed to practice only under supervision and in accordance with their level of education, training and experience (Hamm et al. 2016). Clinical associates reported the following personal professional challenges: lack of independence, insufficient remuneration and opportunities to progress in their career and that their profession only supports physicians. This places additional pressure on the CAs professional relationship with the PNs because the CAs are sidelined by some PNs. Clinical associates’ perception of poor remuneration is valid as Mgobhozi (2019) mentioned that the remuneration of CAs remains low and do not reflect the workload of these associates within the practice environment.

### Strengths and limitations

These results are only the perceptions of the participants in this study and cannot be generalised to all practice environments, PNs and CAs in the South African healthcare sector. Data could be interpreted differently during data analysis by other researchers. However, the authors believe this study provides baseline data to address the identified knowledge gap.

### Recommendations

A larger study with a broader sample and in different provinces, to explore different views and challenges from other HCPs in terms of professional relationships, is recommended. The CAs should have distinguishing devices such as a specific title or name tags. Communication could be improved through meetings and using the standard operating procedure files to discuss the scope of practice of all HCPs, including the new cadre of CAs. Disrespect can be countered by forming a committee of HCPs consisting of members of the multidisciplinary team (Grissinger 2017). In the authors’ opinion, such a committee could draw up a code of professionalism, establish a standard and assertive communication process, train staff, and implement programmes such as conflict management, team building projects and workshops on professionalism. Mutual trust needs to be fostered and cultivated through professional behaviour, education and employee engagement which will create work satisfaction for the HCPs.

## Conclusion

This is the first study that aimed to understand the professional relationship between PNs and CAs in the South African context. In conclusion there are various professional relationship challenges between the two studied populations. However, these challenges could be overcome by a proper introduction of the relatively new clinical associate profession and their specific role in the South African healthcare sector. This could be done through media, workshops, roadshows, in-service training and good communication within various hospitals and departments where CAs are employed.

### Practical implication of the study

By giving support to PNs and CAs in understanding their professional relationship challenges, there will be a multifaceted impact. The impact will be personal and professional morale, interdisciplinary relationships, the practice environment in general and quality care delivered to the community.
